# Occupational exposure to unburnt tobacco and potential risk of toxic optic neuropathy: A cross-sectional study among *beedi* rollers in selected rural areas of coastal Karnataka, India

**DOI:** 10.1371/journal.pone.0188378

**Published:** 2017-11-17

**Authors:** Soujanya Kaup, Ansaba Naseer, Siddharudha Shivalli, Cynthia Arunachalam

**Affiliations:** 1 Department of Ophthalmology, Yenepoya Medical College, Yenepoya University, Mangalore, Karnataka, India; 2 Department of Public Health, Yenepoya Medical College, Yenepoya University, Mangalore, Karnataka, India; 3 Non-Communicable Diseases Regional Technical Advisor, Southeast Asia Regional Office (SEARO), TEPHINET, A Program of The Task Force for Global Health, Inc., Decatur, GA, United States of America; Johns Hopkins University Bloomberg School of Public Health, UNITED STATES

## Abstract

**Background:**

*Beedi* also known as poor man’s cigarette is manufactured in almost all major states of India. *Beedi* workers are exposed to various health risks. There is an increased risk of systemic absorption of tobacco through skin and mucous membrane. The optic nerve is susceptible to damage from several toxic substances including tobacco. This group of disorders is known as toxic optic neuropathy (TON). The association of TON with occupational exposure to unburnt tobacco in *beedi* rollers has not been explored.

**Objectives:**

Among the *beedi* rollers in *Mangaluru* and *Bantwal talukas* of Dakshina Kannada District, Karnataka, India: to assess the magnitude of potential TON utilizing colour vision and contrast sensitivity as screening tools and to identify the demographic, biological and occupational factors associated with potential TON.

**Methods:**

A community-based cross-sectional study was conducted from April-Sept 2016 in *Mangaluru* and *Bantwal talukas*, of *Dakshina Kannada* district, Karnataka. *Beedi* rollers from twelve villages (six from each t*aluka*) were included. In each of the selected villages, the investigators identified *beedi* collection centres and all the eligible *beedi* rollers were included in the study till the required number of *beedi* rollers for that village was achieved. Participants were screened at the study site for visual acuity, colour vision and contrast sensitivity and those with abnormal colour and contrast sensitivity in the presence of good visual acuity were considered to have potential TON.

**Results:**

A total of 377 *beedi* rollers were approached; of which 365 consented to take part in the study (response rate: 96.81%). Women constituted the majority of the participants (n = 338, 92.6%). Based on the screening criteria, the prevalence of potential TON was 17.5% (n = 64, 95% CI: 13.5–21.9). On multiple logistic regression analysis, duration of beedi rolling (Adj OR: 1.061; 95% CI 1.015–1.109, p = 0.009), advancing age (Adj OR: 1.096; 95% CI 1.058–1.136, p<0.001) and presence of diabetes (Adj OR: 6.315; 95% CI 1.4572–27.376, p = 0.014) were independent correlates of potential TON.

**Conclusion:**

In the present study, almost one out of six *beedi* rollers displayed clinical signs of potential TON. Increased duration of *beedi* rolling, advancing age and presence of diabetes were the independent correlates of potential TON. However, with this cross-sectional study it is not possible to conclude if these factors play a role individually or collectively or are a serendipitous association, for which large scale analytical studies are required.

## Background

*Beedi* manufacturing is a traditional home based small scale industry, spread over almost all the major states of India. *Beedi* which is also called poor man’s cigarette accounts for over half of the tobacco consumed in India [1]. It is made of 0.2–0.3 g of tobacco flake wrapped in a *tendu* (*Diospyros melanoxylon*) leaf and secured with coloured thread at both the ends [1]. Within the unorganized household industries, *beedi* sector ranks as the top most employer in India and predominantly employs poor women who hand roll *beedi*s at home to earn a meager but crucial subsistence level income [2]. Although it is estimated that 4.16 million workers are employed in this industry in India, the actual numbers might be as high as 10 million [3–4].

The *beedi* industry not only poses health risks to the *beedi* smoker, but also to the people involved in the *beedi* manufacturing industry. Several studies from India have revealed that *beedi* workers are predisposed to respiratory, dermatological, ophthalmic, and podiatric problems [5–10]. Toxic constituents (i.e. nicotine, nitrosamines, polycyclic aromatic hydrocarbons, formaldehyde, hydrogen etc) present in tobacco are released into the ambient air during processing of *beedi*s. It has been found that inspirable dust in tobacco factory is 150 folds higher than the non-factory settings [11]. The nicotine concentration in the tobacco of *beedi* (21.2 mg/g) is significantly higher than that of commercial filtered (16.3 mg/g) and unfiltered cigarettes (13.5 mg/g) [12]. Nicotine released from the tobacco leaves can be absorbed through skin, respiratory epithelium, and mucous membrane of the mouth [11]. High levels of tobacco constituents (i.e. cotinine, thioethers, promutagens and direct acting mutagens) have been found in *beedi* worker’s urine indicating increased systemic exposure to tobacco [11]. This may result in increased chromosomal aberrations and elevated mutagenic burden among tobacco processors as shown by cytogenetic analysis [13–14].

The optic nerve is susceptible to damage from several toxic substances including tobacco. This group of disorders is known as toxic optic neuropathy (TON) [15]. All individuals, irrespective of age, race, geographic location, and economic strata are at the risk of developing TON. However, certain groups are at higher risk because of occupational exposure to unburnt tobacco or consumption of tobacco and/or other toxic substances or drugs [16]. The association of TON with occupational exposure to unburnt tobacco in *beedi* rollers has not been explored and hence, this study was planned.

The detection of subclinical toxicity is rather difficult in cases of TON [17]. Screening for TON should include a positive history of exposure to the toxic substance (tobacco in this case), signs and symptoms compatible with TON which do not precede the history of exposure [16]. Screening for TON is often difficult because of the non-specific signs and symptoms associated with the disease.

The earliest clinical feature of TON is dyschromatopsia (a change in colour vision). This loss of colour vision is out of proportion to the decline in visual acuity [18]. Acquired colour vision deficit in the presence of good visual acuity strongly suggests optic nerve dysfunction [19]. Also, contrast sensitivity testing has been shown to be an effective clinical tool for detecting subclinical TON as the condition is associated with a decrease in contrast sensitivity [20]. Hence, colour vision and contrast sensitivity can be used for screening of TON.

With this background, this study was planned (among the *beedi* rollers in *Mangaluru* and *Bantwal talukas* of *Dakshina Kannada* District, *Karnataka*, India) with following objectives:

1. To assess the magnitude of potential TON utilizing colour vision and contrast sensitivity as screening tools
2. To identify the demographic, biological and occupational factors associated with potential TON

## Methods

### Study setting

**Fig 1:**
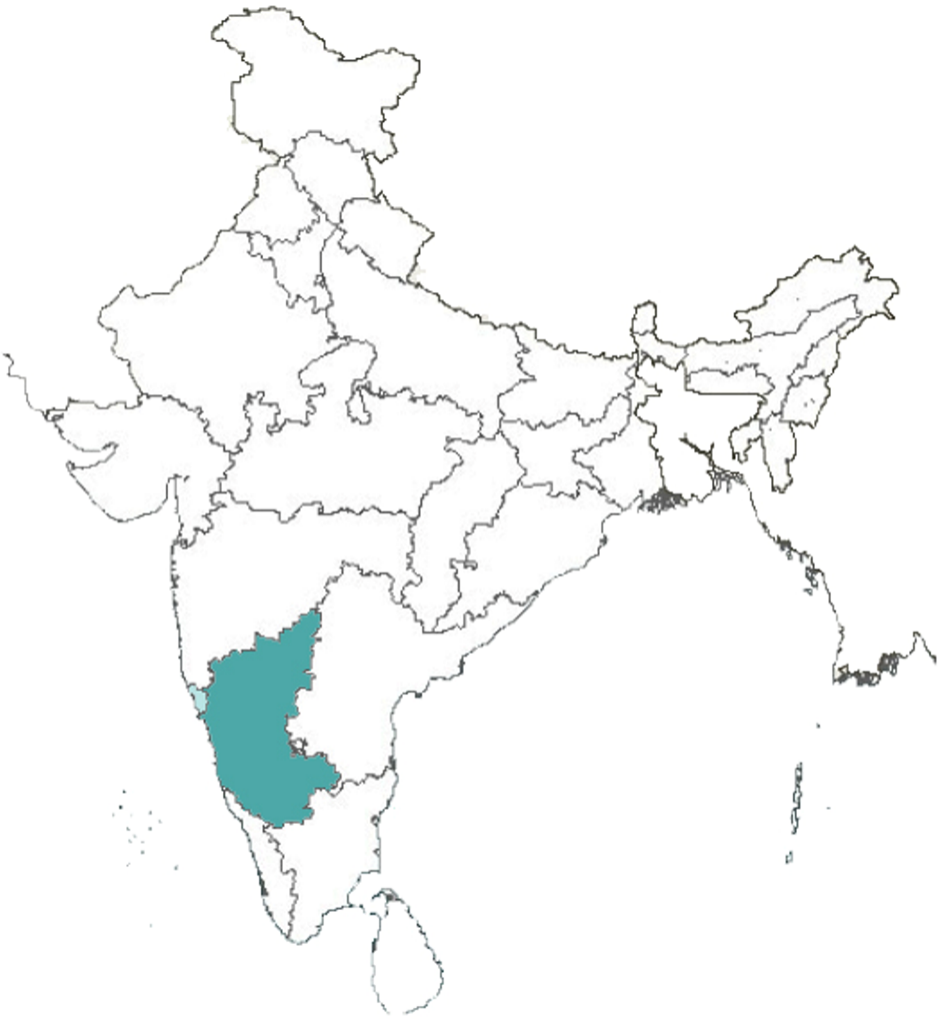
Map of India with Karnataka (highlighted) [Images available from: http://office.incometaxindia.gov.in/bengaluru/Pages/default.aspx].

**Fig 2:**
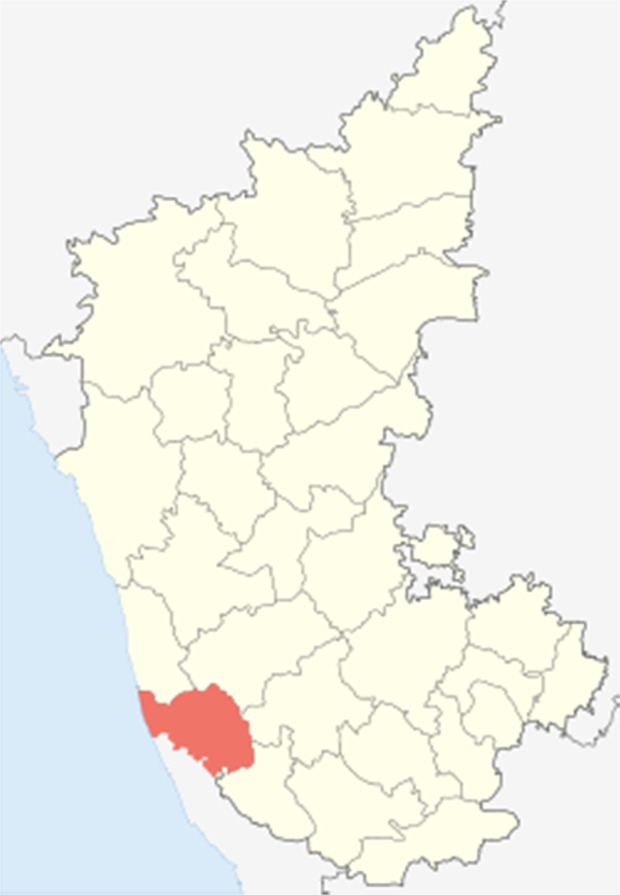
Map of Karnataka with Dakshina Kannada district (highlighted) [Images available from: https://en.wikipedia.org/wiki/Dakshina_Kannada].

*Dakshina Kannada* is a coastal district in the state of Karnataka, India with a total population of 2.09 million [Figs 1 and 2]. The district is divided into 5 *talukas* (an area of the land with a city or town that serves as its administrative centre and a number of villages). Based on the agro-climatic conditions, the district has been divided into coastal (consisting of *Mangalore* and *Bantwal talukas*) and *malnad* regions (consisting of *Puttur*, *Belthangady* and *Sullia talukas*).

### Study design and sample

A community-based cross-sectional study was conducted from April-Sept 2016 in *Mangaluru* and *Bantwal talukas*, of *Dakshina Kannada* district, Karnataka. *Beedi* rollers from twelve villages (six from each t*aluka*) were included. Assuming that 19% of the *beedi* rollers in the study population have TON [21], a sample of 247 was calculated for estimating the expected proportion with 5% absolute precision and 95% confidence. The sample size was inflated to 395 assuming a response rate of 60%.

### Sampling

*Beedi* rolling is an unorganised small scale home-based industry. Therefore, drawing a community based representative sample is difficult. The investigators contacted the *beedi* contractors and enlisted the villages where *beedi* rolling was prevalent in the two study *talukas*. Keeping the resource constraints in mind, it was decided to include a total of twelve villages (six in each *taluka*). The number of *beedi* rollers from each village to be included in the study was based on proportional allocation. In each of the selected village, the investigators identified *beedi* collection centres and all the eligible *beedi* rollers coming to these *beedi* collection centres were included in the study.

### Inclusion and exclusion criteria

Inclusion criteria:

1. Person involved in *beedi* rolling for at least one year.
2. Person willing to participate in the study

Exclusion criteria:

1. Persons with significant ocular media opacity hindering fundus evaluation.
2. History of prior significant ocular or head trauma
3. Persons on any of the following medications known to cause TON, like: chloramphenicol, sulfonamides, linezolid, Chloroquine, quinine, Isoniazid, ethambutol, streptomycin, Digitalis, amiodarone, Vincristine and methotrexate.

### Data collection and analysis

*Beedi* rollers engaged in *beedi* rolling for at least one year were considered to be exposed to unburnt tobacco. A pre-tested semi-structured interview schedule was used to elicit the data on age, gender, economic status, systemic co-morbidities (diabetes and hypertension), ocular symptoms, and tobacco consumption in any form. The type of ration card possessed by the *beedi* roller was considered to determine their economic status.

They were screened at the study site for visual acuity, colour vision and contrast sensitivity. Farnsworth D15 (Richmond Products Inc. Albuqueeque, New Mexico) test was used for testing colour vision [22]. The D15 set is a modification of the well-known Farnsworth-Munsell 100 Hue Test. Each D15 set contains a reference disc and fifteen numbered discs, which make up an incomplete colour circle. Following an attempt to sequentially arrange the discs by the patient, evaluation determines colour perception or defects in deutan, protan or tritan axis discrimination. Sometimes there are indeterminate defects in the cases of retinal toxicity. Scoring was accomplished by reading the colour chip numbers on the reverse side and recording the sequence selected by the patient on a copy of score sheet. A patient with a colour vision deficiency will arrange the colour discs in a different order than a person with normal colour vision. A line is then drawn from the starting point (reference disc) through the sequence determined by the participant. Presence of colour vision defect was determined if the sequence lines crossed the centre repeatedly [22]. Test was repeated for the participants with subnormal results.

Pelli-Robson chart [23] was used to assess the contrast sensitivity. It consists of letters of the same size but with reducing contrast. Each chart has six letters in each row organised into two triplets of varying contrast. The illumination of 85 cd/mm^2^ is required for the chart. It must be used at 1 meter distance. The score of the test was recorded by the faintest triplet out of which at least 2 letters are correctly identified. The log contrast sensitivity value for this triplet is given by the number on the scoring pad. Participants with abnormal colour and contrast sensitivity in the presence of good visual acuity were labelled as potential TON. Questionnaire administration and screening for potential TON were done by two people independently.

Data were analyzed using Statistical Package for the Social Sciences (SPSS) for Windows, Version 16.0. Chicago, SPSS Inc. Results were expressed as frequencies and proportions for categorical variables and mean and standard deviations for continuous variables. Chi-square test was applied to assess the differences in potential TON across various study variables. A two-sided p<0.05 was considered as statistically significant. Multivariable logistic regression was applied to explore the independent correlates of potential TON. Proportion and adjusted Odds ratio (adjOR) with 95% confidence intervals for potential TON were the key outcome measures.

### Ethical approval

The institutional review board and the ethics committee of Yenepoya University approved the study protocol (YUEC223/2016, Date: 21^st^ April 2016). Informed written consent in local language (*Kannada)* was administered to all the study participants for voluntary participation. In case of illiterate participant, details of the study were explained to in the presence of a witness and left thumb impression of the participant and the signature of the witness were taken on the consent form.

## Results

A total of 377 *beedi* rollers were approached and 365 consented to take part in the study (response rate was 96.81%). As many as 338 (92.6%) study participants were women. Observed gender wise difference in the mean ages (female: 43.7±12.69 years vs. males: 43.89 ± 10 years) was not statistically significant (t = 0.076, p = 0.939) [Table 1].

**Table 1:** Descriptive statistics of *beedi* rollers in selected rural areas of *Dakshina Kannada* district, Karnataka, India, April-Sept 2016 (N = 365).

Study variable	N	%
**Age (years)**		
20–30	74	20.3
31–40	69	18.9
41–50	130	35.6
51–60	48	13.2
>60	44	12.1
**Gender**		
Male	27	7.4
Female	338	92.6
**Economic status**		
Below poverty line	311	85.2
Above poverty line	54	14.8
**Ocular symptoms**		
Blurred vision	106	29
Headache	42	11.5
Irritation	25	6.8
Watering	21	5.8
Eye pain	6	1.6
Redness	4	1.1
Giddiness	4	1.1
Discharge	2	0.5
**Systemic co-morbidities**		
Hypertension	33	9
Diabetes Mellitus	12	3.3
**Tobacco consumption (in any form)**	17	4.7

Both males and females were employed for almost an equal mean number of years (F = 15.77 ± 8.82yrs, M = 16.44 ± 9.64yrs; p = 0 .820). Male participants worked for significantly more number of hours per day as compared to females (p = 0.003). Male participants rolled more number of *beedi*s per day than females (p<0.001). *Beedi* rollers were on an average engaged in *beedi* rolling for 15.82 ± 8.87 years. Their mean daily working period was 4.19± 1.47 hours/day and 462.14± 281.27 *beedi*s were rolled in a day.

Based on the screening criteria (abnormal colour vision and reduced contrast sensitivity in the presence of good visual acuity), the prevalence of potential TON was 17.5% (n = 64, 95% CI: 13.5–21.9).

On bivariate analysis, tobacco intake and diabetes mellitus were found to have a significant association (p<0.05) with potential TON [Table 2]. Mean age (56.83±10.65 vs. 40.9±11.02, t = 10.78, p<0.01) duration of beedi rolling (24.02 ± 9.402 vs. 14.08±7.724, t = 7.9, p<0.01) and number of beedis rolled per day (525.78±287.572 vs.448.6±278.531, t = 2, p = 0.011) were significantly higher among those with potential TON when compared to those without potential TON [Table 3].

**Table 2:** Association between key demographic, biological, and occupational factors, and potential toxic optic neuropathy among *beedi* rollers in selected rural areas of *Dakshina Kannada* district, Karnataka, India, April-Sept 2016 (N = 365).

Study variable	Possible TON		Total	χ^2^	p value
	No	Yes			
**Gender**					
Female	277 (82.0%)	61 (18.0%)	338 (100.0%)	0.8	0.443
Male	24 (88.9%)	3 (11.1%)	27 (100.0%)		
**Tobacco intake**					
No	291 (83.6%)	57 (16.4%)	348 (100.0%)	6.8	0.017
Yes	10 (58.8%)	7 (41.2%)	17 (100.0%)		
**Diabetes**					
No	298 (84.4%)	55 (15.6%)	353 (100.0%)	28.3	<0.001
Yes	3 (25.0%)	9 (75.0%)	12 (100.0%)		
**Hypertension**					
No	277 (83.4%)	55 (16.6%)	332 (100.0%)	2.38	0.123
Yes	24 (72.7%)	9 (27.3%)	33 (100.0%)		
**History of** **tuberculosis**					
Absent	300 (82.4%)	64 (17.6%)	364 (100.0%)	0.2	1.0
Present	1 (100.0%)	0 (0%)	1 (100.0%)		

**Table 3:** Comparison of mean age, duration of *beedi* rolling, working hours per day and daily number of *beedis* rolled between those with and without potential toxic optic neuropathy (TON) among *beedi* rollers in selected rural areas of *Dakshina Kannada* district, Karnataka, India, April-Sept 2016 (N = 365).

Study variable	Potential TON (n = 64)		No Potential TON (n = 301)		t	p
	Mean	SD	Mean	SD		
Age	56.83	10.65	40.92	11.02	10.78282	<0.001
Duration of *beedi* rolling	24.02	9.402	14.08	7.724	7.9	<0.001
Working hours per day	4.05	1.786	4.22	1.404	-0.712	0.478
Daily number of *beedi*s rolled	525.78	287.572	448.6	278.531	2	0.011*

* Mann-Whitney U test

On multiple logistic regression analysis, duration of beedi rolling (Adj OR: 1.061; 95% CI 1.015–1.109, p = 0.009), age (Adj OR: 1.096; 95% CI 1.058–1.136, p<0.001) and presence of diabetes (Adj OR: 6.315; 95% CI 1.4572–27.376, p = 0.014) were the independent correlates of potential TON [Table 4]. According to regression analysis, the prevalence of potential TON increases by 9.6% and 6.1% for each additional year of age and each additional year of beedi rolling, respectively. Similarly, the odds of potential TON increases by 6.3 times if the beedi roller has diabetes.

**Table 4:** Multiple logistic regression analysis for various factors associated with potential toxic optic neuropathy among *beedi* rollers in selected rural areas of *Dakshina Kannada* district, Karnataka, India, April-Sept 2016 (N = 365).

Variables of potential TON	Adj OR	95.0% C.I.		P value
		Lower	Upper	
Diabetes	6.315	1.457	27.376	**0.014**
Tobacco consumption	1.540	0.432	5.487	0.506
Age	1.096	1.058	1.136	**<0.001**
Duration of *beedi* rolling	1.061	1.015	1.109	**0.009**
Daily number of *beedi*s rolled	1.000	0.999	1.001	0.994

## Discussion

In TON, dyschromatopsia is often the earliest symptom [18]. Acquired colour vision deficit in the presence of good visual acuity strongly suggests optic nerve dysfunction [19]. Contrast sensitivity testing has also been shown to be an effective method for detecting subclinical TON [20]. Hence, in the present study, we screened *beedi* rollers for dyschromatopsia (tested by Farnsworth D 15 test) and reduced contrast sensitivity (tested on Pelli-Robson chart). Based on the screening criteria, 17.5% (n = 64) participants were detected to have potential TON. *Mithal S et al*. (2008), found 19% of *beedi* rollers to have associated optic neuropathy [21]. Silvette et al. in an extensive review calculated the incidence of pure tobacco optic neuropathy in a population of nearly 300,000 patients with eye disease as 0.77% [24]. In the present study, the reported proportion of potential TON was much higher than in general population.

In the present study, three associations were found with potential TON: duration of *beedi* rolling, increasing age and presence of diabetes mellitus. Longer duration of b*eedi* rolling probably results in increased absorption of tobacco constituents through the skin, respiratory epithelium, and mucous membrane [11], which over the years may lead to tobacco-related TON.

In the present study, advancing age was also an independent correlate of potential TON. One may debate that ageing process itself may have resulted in abnormal colour vision. Available evidence suggest that in the general population only 5–7% of the individuals aged 60 to 70 years and about 10% of the 70 to 75 year age group fail the Farnsworth D-15 test [25]. However, in the present study, 68.2% (n = 30 out of 44) of the individuals aged ≥60 years failed the Farnsworth D-15 test, which cannot be attributed to ageing alone.

Metabolic diseases, including diabetes mellitus, might influence TON due to the accumulation of toxins [15]. A similar association was found in the present study.

Although the screening tests (colour vision and contrast sensitivity) used for detection of TON are sensitive, they are not specific. Old age and diabetes can also result in abnormal colour vision and contrast [26, 27]. But, an acquired abnormal colour vision in the presence of good visual acuity points towards optic neuropathy [19]. In the present study too, participants with abnormal colour vision and decreased contrast in the presence of good visual acuity were labeled as potential TON.

### Strength and limitations

This study had more than expected response rate for screening potential TON (96.81%).The study did not collect any sensitive information and the investigators went to the community for data collection. Also, the screening procedure was quick and simple. Hence, more than expected response rate was observed.

Diagnosis of potential TON was based on screening tests only. TON could be confirmed only in one patient with visual evoked potential, as most of the patients did not return to the base hospital even on repeated requests. Social and economic barriers prevented the participants from seeking further medical attention even though it was made freely available. *Beedi* rollers tend to lose a day’s wage as a result of hospital visit and hence patients chose not to attend the hospital for further investigations since they continued to enjoy good visual acuity. The observed association between occupational exposure to unburnt tobacco and potential TON may not be causal owing to cross-sectional nature of the study. Similarly, diabetes could influence the occurrence of potential TON, but causal association cannot be established with this study design. In general, poor health and nutritional deficiency might have played a role in occurrence of optic neuropathy which could not be assessed in this study.

## Conclusion

Almost one out of six *beedi* rollers in the present study displayed clinical signs of potential TON. Longer duration of *beedi* rolling, advancing age and presence of diabetes were independent correlates of potential TON. However, with this cross-sectional study it is not possible to conclude if these factors play a role individually or collectively or are a serendipitous association, for which large scale analytical studies are required.

### Recommendations

*Beedi* rollers must be enlightened on the harmful effects of tobacco before enrolling them in this industry. Failure to do so can also open the industry to litigation. They must be screened for TON on a regular basis as they may be symptom free for prolonged periods. Special emphasis must be given to patients who are older, engaged in *beedi* rolling for longer duration and who have diabetes mellitus. Precautions like wearing gloves, mask, frequent hand washing must be encouraged to decrease systemic absorption of tobacco through skin and mucosa.

## Supporting information

S1 STROBE Checklist (DOC)

S1 Data Sheet (XLSX)
